# miR-100-5p Downregulates mTOR to Suppress the Proliferation, Migration, and Invasion of Prostate Cancer Cells

**DOI:** 10.3389/fonc.2020.578948

**Published:** 2020-12-01

**Authors:** Yun Ye, Su-Liang Li, Jian-Jun Wang

**Affiliations:** ^1^ Department of Laboratory Medicine, The First Affiliated Hospital of Xi’an Medical University, Xi’an, China; ^2^ Emergency Department, The First Affiliated Hospital of Xi’an Medical University, Xi’an, China

**Keywords:** prostate cancer cell, miR-100-5p, mTOR, downregulates, LNCaP

## Abstract

**Background:**

Previous studies have shown that miR-100-5p expression is abnormal in prostate cancer. However, the role and regulatory mechanism of miR-100-5p requires further investigation. Thus, the aim of this study was to observe the effects of miR-100-5p on the proliferation, migration and invasion of prostate cancer (PCa) cells and to explore the potential related regulatory mechanism.

**Materials and Methods:**

Differential miRNA expression analysis was performed using next-generation sequencing (NGS) in the patients with PCa and benign prostatic hyperplasia (BPH). The expression levels of miR-100-5p were detected using real-time fluorescence quantitative PCR (qRT-PCR). PCa cells were transfected with NC-mimics or miR-100-5p mimics, inhibitor by using liposome transfection. Moreover, the CCK-8 proliferation assay, colony formation assay, cell scratch assay and Transwell assay were used to detect the effects of miR-100-5p on cell proliferation, migration, and invasion. In addition, the target gene of miR-100-5p was verified by luciferase reporter gene assay, and the influence of miR-100-5p on the expression of mTOR mRNA by qRT-PCR and the expression of mammalian target of rapamycin (mTOR) protein was detected by western blot and immunohistochemical staining.

**Results:**

Differential expression analysis of high-throughput sequencing data showed low expression of miR-100-5p in the patients of PCa. It was further confirmed by qRT-PCR that the expression of miR-100-5p in PCa cells was significantly lower than that in RWPE-1 cells (*P*<0.01). miR-100-5p expression in lymph node carcinoma of prostate(LNCaP) cells was markedly upregulated after transfection with miR-100-5p mimics (*P*<0.01), while cell proliferation, migration and invasion capacities were clearly reduced (*P*<0.01). mTOR mRNA and protein expression was also substantially lowered (*P*<0.01) and mTOR adjusted the expression of NOX4. Finally, we further confirmed by immunohistochemical staining that miR-100-5p regulated the expression of mTOR and NOX4.

**Conclusion:**

miR-100-5p is expressed at low levels in PCa cells, and it can suppress PCa cell proliferation, migration and invasion, the mechanism of which is related to downregulating the expression of mTOR.

## Introduction

PCa is the most common malignant tumor of the male reproductive system. According to the latest statistics, a projected 191,930 new cases of PCa will be diagnosed, accounting for more than one in five new diagnoses of male tumors. An estimated 33,330 men may die of the disease in the United States in 2020, and mortality due to PCa accounts for 10% of all cancer deaths (1). With an increasingly aging population and changes in diet, the incidence of prostate cancer in Asia is increasing year by year (2). MicroRNAs (miRNAs) are highly conserved noncoding single-stranded RNAs consisting of 21–24 nucleotides that regulate the expression of target genes through complete or incomplete complementarity binding with target genes and play an important role in the gene regulatory network (3, 4). Recent studies have found that miRNAs, as oncogenes or tumor suppressor genes, play an important role in the development and progression of malignant tumors (5). A large number of studies have confirmed that miRNA expression is dysregulated in the occurrence and development of prostate cancer, and there is differential expression between prostate cancer patients and the normal population (6, 7).

MiR-100-5p is an important member of the miRNA family, located on chromosome 11q24.1 and highly conserved. MiR-100-5p has abnormal expression in many malignant tumors, including prostate cancer and is involved in biological behaviors such as proliferation, migration, and invasion of tumor cells (8). Our previous work found that the levels of miR-100-5p from LNCaP cells were significantly lower than those in the other cell lines (9). In this study, we observed changes in the expression of miR-100-5p in the PCa patients and cell lines and its effect on cell proliferation, migration and invasion ability, and explored its potential molecular mechanism.

## Materials and Methods

### Patients and Blood Samples

Patients initially diagnosed with PCa at The First Affiliated Hospital of Xi’an Medical University (Xi’an, People’s Republic of China) between January 2015 and October 2019 were included in the PCa group. Age- and sex-matched patients with diagnosed BPH were collected as a control group. Blood sampling and examination were performed prior to treatment, including surgery, radiotherapy, or chemotherapy. The peripheral blood was collected into serum-separator tubes and allowed to stand for 1 h at room temperature, prior to centrifugation at 3,000× g for 10 min. The resulting serum was transferred into fresh tubes and stored at -80°C for further analysis. The present study was approved by the Medical Ethics Committee of The First Affiliated Hospital of Xi’an Medical University, and written informed consent was obtained from all participants (No. xyfy2015011).

### Cell Culture

LNCaP, PC-3, DU-145 (PCa cell line), and RWPE-1 (normal prostatic epithelial cells) were purchased from American Type Culture Collection (Manassas, VA, USA). LNCaP, PC-3, and DU-145 cells were grown separately in Roswell Park Memorial Institute (RPMI)-1640 (Gibco; Thermo Fisher Scientiﬁc, Inc., Waltham, MA, USA) and RWPE-1 cells were grown in Keratinocyte Serum Free Medium (K-SFM) (Gibco BRL Co. Ltd., USA). All culture media were supplemented with 10% fetal bovine serum (Gibco; Thermo Fisher Scientiﬁc), 100 U/ml penicillin and 100 mg/ml streptomycin (HyClone; GE Healthcare Life Sciences). All cells were routinely cultured in a humidity incubator at 37°C and 5% CO2, and logarithmic-phase cells with good growth conditions were selected for subsequent experiments. The cells tested negative for mycoplasma contamination, and this testing was completed every 3 months and after the initiation of cell culture.

### Construction of the cDNA Library

MiRNAs were isolated and purified by gel electrophoresis, and cDNA was synthesized by reverse transcription. The miRNA sequencing library was obtained by PCR amplification. cDNA libraries were tested using an Illumina Hi Seq 2500 sequencer (GUANGZHOU RIBOBIO CO., LTD). The protocol for construction of the library: TotalRNA was isolated using the TRIzol reagent (Invitrogen;Thermo Fisher Scientific, Inc.) according to the manufacturer’s protocol. RNA purity was assessed using the ND-1000 Nanodrop. RNA integrity was evaluated using the Agilent2200 TapeStation (Agilent Technologies, Inc., Santa Clara, CA, USA) and each sample had a RINe >7.0. Brieﬂy, RNAs were ligated with 3’RNA adapter, followed by 5’adapter ligation. Subsequently, the adapter-ligated RNAs were subjected to RT−PCR and ampliﬁed with a low−cycle. The PCR products were size selected by PAGE gel according to instructions of NEBNext^®^Multiplex. The original 50 nt raw reads were preliminarily ﬁltered, and clean reads were obtained through Illumina HiSeq™ 2500 sequencing (Illumina, Inc., San Diego, CA, USA). Distribution of sequence length and the consensus sequence of the sample were calculated statistically. Clean reads were classiﬁed and annotated to obtain the composition and expression information of various small RNAs in the samples.

### Differential Expression Analysis of miRNA

miRNA sequences were compared with known human miRNA sequences from the miRBase 21.0 database (http://www.mirbase.org/). Scatter plots and log2 ratios were used to compare the co-expression of miRNAs. Edger analysis was used to analyze the significance of differences in miRNA expression in each group and calculate the P value, for which the -log10p value was calculated and the differentially expressed miRNAs were screened. R Studio1.1.463-Windows Vista/7/8/10 software was used to perform cluster analyses.

### RNA Preparation and RT-PCR

We isolated total RNA and total miRNA from cells by using TRIzol (Life Technologies) reagent and the mirVana miRNA Isolation Kit (Ambion). We treated total RNA with TURBO DNase (Ambion) after purification and used a high-capacity cDNA reverse transcription kit (Takara) to reverse transcribe RNA into first-strand cDNA. We purchased primers against mRNAs from Shanghai Sangon Biotech Co., Ltd. Each experiment was carried out in triplicate, and the mean value of the three-cycle threshold was used for further analysis. Cel-miR-39 was used as an internal control. The expression values of miRNAs were normalized to the U6 values, and the relative quantification analysis was performed using the 2^-ΔΔCt^ method. The primers for miR-100-5p were F: 5’-GAACCCGTAGATCCGAACT-3’ and R: 5’-CAGTGCGTGTCGTGGAGT-3’. The primers for U6 were F: 5’-CTCGCTTCGGCAGCACA-3’ and R: 5’-AACGCTTCACGAATTTGCGT-3’. The primers for mTOR were F: 5’-CTGGGACTCAAATG TGTGCAGTTC-3’ and R: 5’-GAACAATAGGGTGAATGATCCGGG-3’. The primers for NOX4 were F: 5’-CAGGAGGGCTGCTGAACTATCAA-3’ and R: 5’-TGACTGGCTTATTGC TCCGGATA-3’.The primers for β-actin were F: 5’-TCCTCCCTGG AGAAGAGCTA-3’ and R: 5’-TCAGGAGGAGCAATG ATCTTG-3’. The amplification conditions were 95°C for 10 min, followed by 40 amplification cycles of 95°C for 10 s and 60°C for 30 s. The ABI Prism 7500 Sequence Detection System (Applied Biosystems) was used to perform real-time PCR.

### Cell Transfection

miR-100-5p mimics, miR-100-5p inhibitor and the miRNA negative control (miR-NC) were synthesized by RIBOBIO Co., Ltd. (Guangzhou, China) and transfected into cells by Lipofectamine 2000 (Invitrogen, New York, USA) according to the manufacturer’s specifications. Cells were transfected at a multiplicity of infection (MOI) of 100 according to the manufacturer’s instructions. The results were examined by qRT-PCR, and further experiments were also carried out after transfection for 48 h.

### Cell Proliferation Assay

The cells were proportionally diluted with culture medium and the OD value was measured after adding Cell Counting kit-8 (CCK-8) (Cat. No.: HY-K0501,MedChemExpress USA) reagent for 8 h 37°C, in order to produce a standard curve. The cell suspension (5000 cells/100 µl/well) was inoculated in a 96-well plate, and 10 µl CCK-8 solution was added to each well. The sample was incubated for 4 h 37°C, and the OD value was measured at 450 nm. The experiments were repeated 3 times.

### Colony Formation Assay

Five-hundred cells were seeded in six-well plates after transfected the cells LNCaP and PC-3 by (miR-100-5p-mimic, miR-100-5-inhibitor, and N.C). After incubation for 2 weeks, colonies were fixed by ethanol for 15 min, followed by staining with crystal violet for 20 min then formative colonies were counted.

### Cell Scratch Assay

PCa cells (5 × 10^3^ cells/well) were cultured in a 24-well plate in RPMI-1640 culture medium with 10% FBS. The culture plate was incubated overnight at 37°C in a humidified CO^2^ incubator. Next, the culture medium was removed, and the adherent cell layer was scratched with a sterile 200 μl pipette tip. We washed away the cell fragments with phosphate-buffered saline (PBS). Images of the scratch area at 0 h and 24 h were taken using a built-in camera in the microscope (40x magnification). Data were evaluated using TScratch imaging software (CSE Lab., ETH, Zurich) to calculate the percent wound area (10).

### Transwell Assay

The Transwell chamber was treated with Matrigel (Solarbio, Beijing, China). After transfection, 2 × 10^5^ PCa cells and N.C control cells were cultured in the upper chamber (Solarbio, Beijing, China) with serum-free medium. RPMI-1640 medium containing 10% FBS was added to the lower chamber, and the Transwell chamber was removed after continued culture for 24 h and maintained at 37°C in 5% CO2 overnight. Subsequently, cells were fixed with methanol for 10 min, stained with crystal violet (Yuanye, Shanghai, China) and then analyzed by microscopy. The numbers of migrated cells were observed from digital images captured on an Olympus microscope (Olympus Inc.) and calculated using Image J software, and the experiment was repeated three times.

### Western Blotting

The cell harvesting and extraction of proteins followed the method described previously (11). A protein assay kit (Bio-Rad, Hercules, CA, USA) was used to determine protein concentrations, and 2 mg/ml samples were loaded onto 10% SDS-PAGE gels to separate the samples according to their molecular weight. PVDF membranes was blocked with TBST buffer (0.05% Tween-20) at room temperature for 60 min. The blotted membrane was incubated with rabbit monoclonal anti-mTOR (1:1,000; ab32028; Abcam), anti-NOX4 (1:1,000; ab133303; Abcam), β-actin (1:1,000; ab8227; Abcam) which was used as a loading control and horseradish peroxidase (HRP)-coupled goat anti-rabbit IgG H&L (1:5,000; ab97051; Abcam) as a secondary antibody. Primary antibody dilutions were incubated with the membranes overnight at 4°C. Then the secondary antibodies were washed by TBS-T-20-Tween six times each time 5 min. The membranes were exposed by chemiluminescence instrument according to the manufacturer’s instructions and followed exposure to ImageQuant LAS 500 (GE Healthcare Life Sciences, Shanghai, China) for 2–12 min. After western blot, the quantification protein expression was measured by using Image J software (International Institute of Health, USA).

### Prediction of Targeted Relationship

MiRGator 3.0 software and TargetScan Human 7.1 were used to predict the targeting relationship between miR-100-5p and mTOR.

### Luciferase Reporter Assay

The dual-luciferase reporter plasmid fused with the wild-type or mutant 3′-UTR segment of human mTOR was obtained from Gene-Pharma (Shanghai, China). For confirmed of the regulation of mTOR by miR-100-5p-mimic, LNCaP, and PC-3 cells transfected with (miR-100-5p-mimic or N.C) were plated into 24-well plates. Followed co-transfected the cells with 0.1 ng of the mTOR wild-type or mutant 3′-UTR reporter plasmids, and 20 nM miR-100-5p-mimic or N.C with Lipofectamine 2000 reagent (Invitrogen, Carlsbad, CA, USA). After 48 h, the cells were harvested with passive lysis buffer and the luciferase activity was calculated by the Dual-Luciferase Reporter Assay System (Promega, Madison, WI, USA) according to the manufacturer’s instructions.

### Tumorigenesis Studies of Animals

All experimental procedures were approved by the Committees of Animal Ethics and Experimental Safety of The First Affiliated Hospital of Xi’an Medical University and followed the Laboratory Animal Management and Welfare (XYFYLA-003)(Specific approval number for work on animals=20). Six-week-old male nude mice were divided into two groups and administered by intraosseous injected with 2.0 × 10^6^ tumor cells in 50% Matrigel™ (Falcon, NJ, USA), LNCaP cells transfected with miR-100-5p mimics (A group) and N.C (B group, control). Pathological staining and immunohistochemical staining were performed after 2 months.

### Immunohistochemical Analysis

Paraffin-embedded tissues were dewaxed, hydrated with ethanol, and incubated with 0.3% H_2_O_2_ to eliminate endogenous peroxidase activity. The primary antibody was added and incubated at 4°C overnight, and the secondary antibody was incubated at room temperature for 1 h. The immunohistochemical results were observed by a Nikon Eclipse 80i microscope, and positive results for brown granules were mainly distributed in the cytoplasm. Positive expression was quantitatively analyzed by Image-ProPlus 5.0 analysis software, and the integral optical density (IOD) value of positive staining in each visual field was calculated. The mean value of IOD of five visual fields was taken as the final value of each group.

### Statistical Analyses

SPSS 20.0 software (SPSS Inc., Chicago, IL, USA) for Windows was used to perform all statistical analyses. The data are presented as the mean ± SD. Nonparametric data were analyzed by 2-tailed Mann-Whitney U-tests. P<0.05 was selected to indicate a statistically signiﬁcant difference.

## Results

### The Expression of miR-100-5p in Serum and Cell Lines

**Figure 1:**
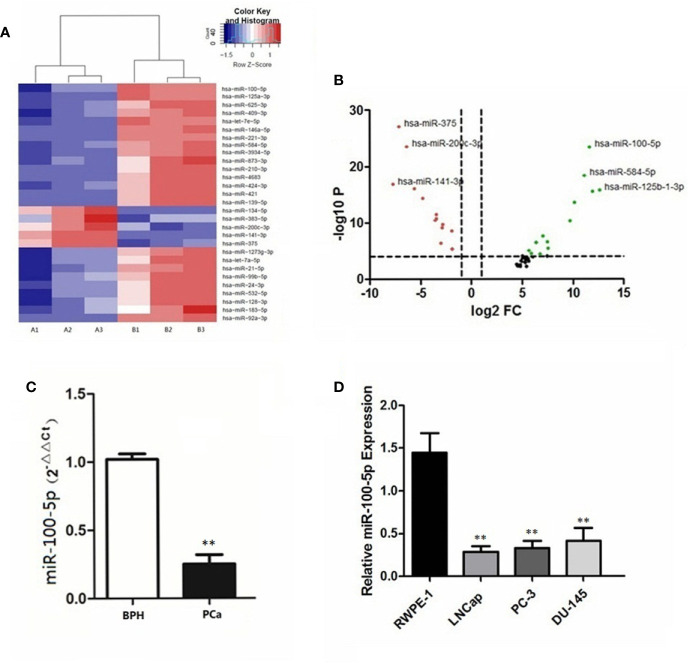
Differential expression of miR−100-5p. **(A)** Heatmap of differences in miRNA expression levels. Red, upregulated; blue, downregulated. Cut−off: |log2 (fold -change) |≥1. A1, A2, A3: PCa; B1, B2, B3: BPH. **(B)** Volcano plot of differences in miRNA expression levels. Red, upregulated; black, intermediate value; green, downregulated. **(C)** The expression of miR-100-5p in serum specimens. **(D)** The expression of miR-100-5p in cell lines. (**P < 0.01).

Compared with patients with BPH in serum sample, 29 miRNAs in patients with prostate cancer were significantly differentially expressed (**Figure 1A**). Notably, miR-100-5p, miR-584-5p and miR-125b-1-3p were downregulated, whereas miR-375, miR-200c-3p, and miR-141-3p expression levels were upregulated (**Figure 1B**). The qRT-PCR results revealed the expression levels of serum miR-100-5p in patients with prostate cancer were significantly lower compared with the BPH group (P<0.01) (**Figure 1C**). The qRT-PCR results further confirmed that the expression of miR-100-5p in prostate cancer cells lines was significantly lower than that in RWPE-1 cells (P<0.01) (**Figure 1D**). The cells LNCaP and PC-3 were selected for further cellular study.

### Expression of miR-100-5p in PCa Cells After Transfection

**Figure 2:**
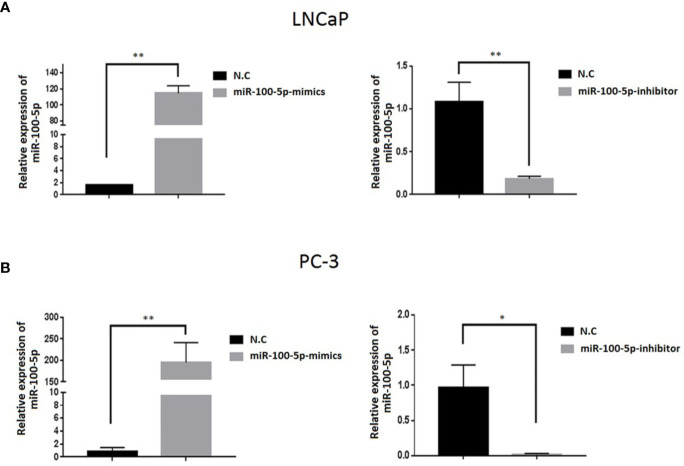
Expression level of miR-100-5p after transfection. **(A, B)** miR-100-5p levels were increased and decreased in LNCaP and PC-3 cells transfected with miR-100-5p-mimics and miR-100-5p-inhibitor.(*P < 0.05 and **P < 0 .01).

The expression of miR-100-5p in the mimics group was significantly higher than that in the NC-mimics group, and miR-100-5p in the inhibitor group was significantly lower than that in the NC-mimics group (P<0.01) (**Figure 2**). This confirmed that miR-100-5p-mimic and miR-100-5p-inhibitor can effectively regulate the expression in prostate cancer cells as shown respectively.

### Proliferation Activity, Migration Capacity, and Invasion Ability of PCa Cells After Transfection

**Figure 3:**
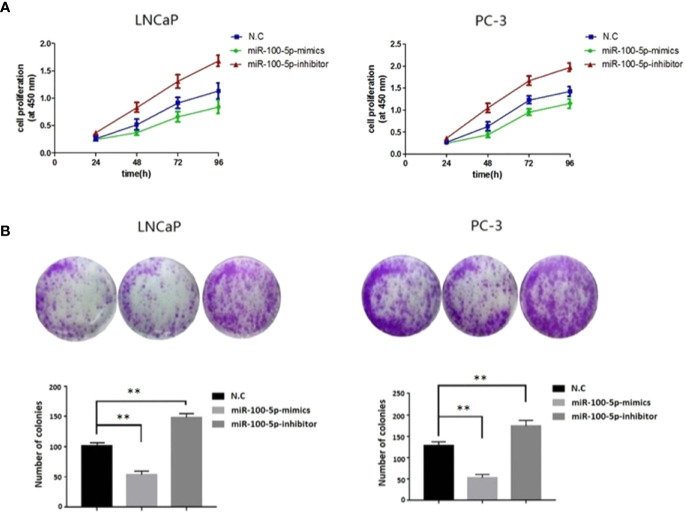
Cell proliferation activity after transfection. **(A)** LNCaP and PC-3 cells were transfected with miR-100-5p-mimics, inhibitor, or N.C for 0, 24, 48, 72, and 96 h. The cell proliferation was performed by CCK-8. **(B)** Colony formation assay of the changes in proliferation capacity of LNCaP and PC-3 cells after transfection with miR-100-5p-mimics, inhibitor, or N.C for 14 days. (**P < 0.01).

**Figure 4:**
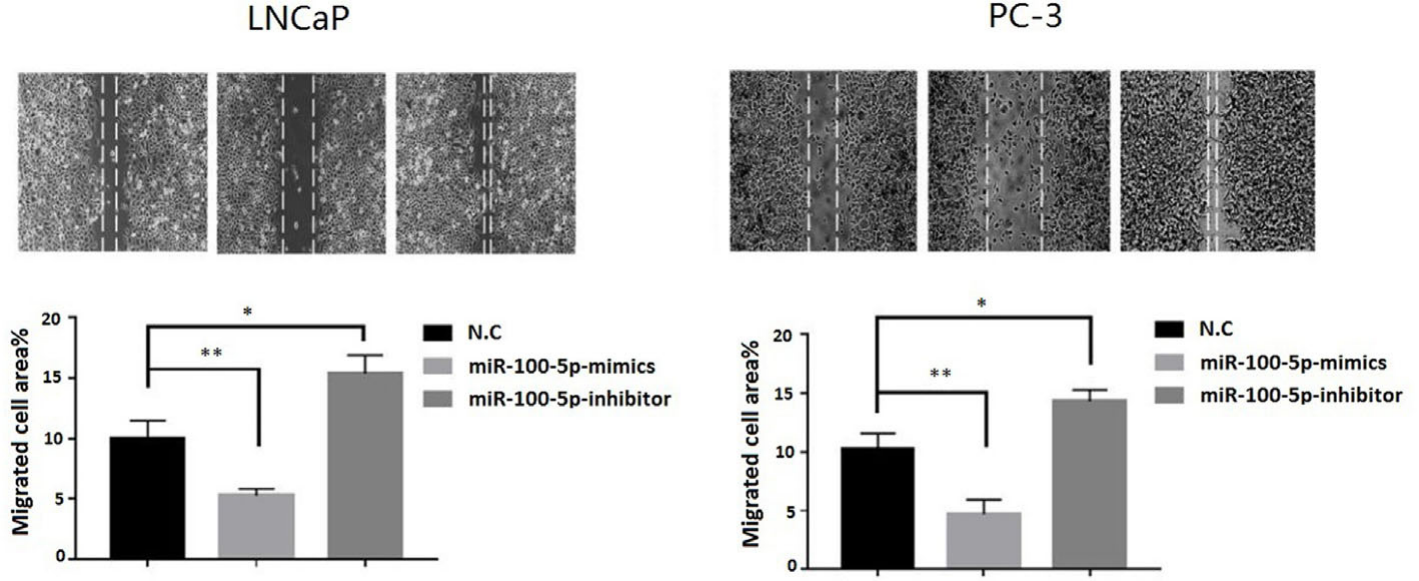
Cell migration ability detected by cell scratch experiment. LNCaP and PC-3 cells were transfected with miR-100-5p-mimics, inhibitor, or N.C for 48 h. (*P < 0.05 and **P < 0.01).

**Figure 5:**
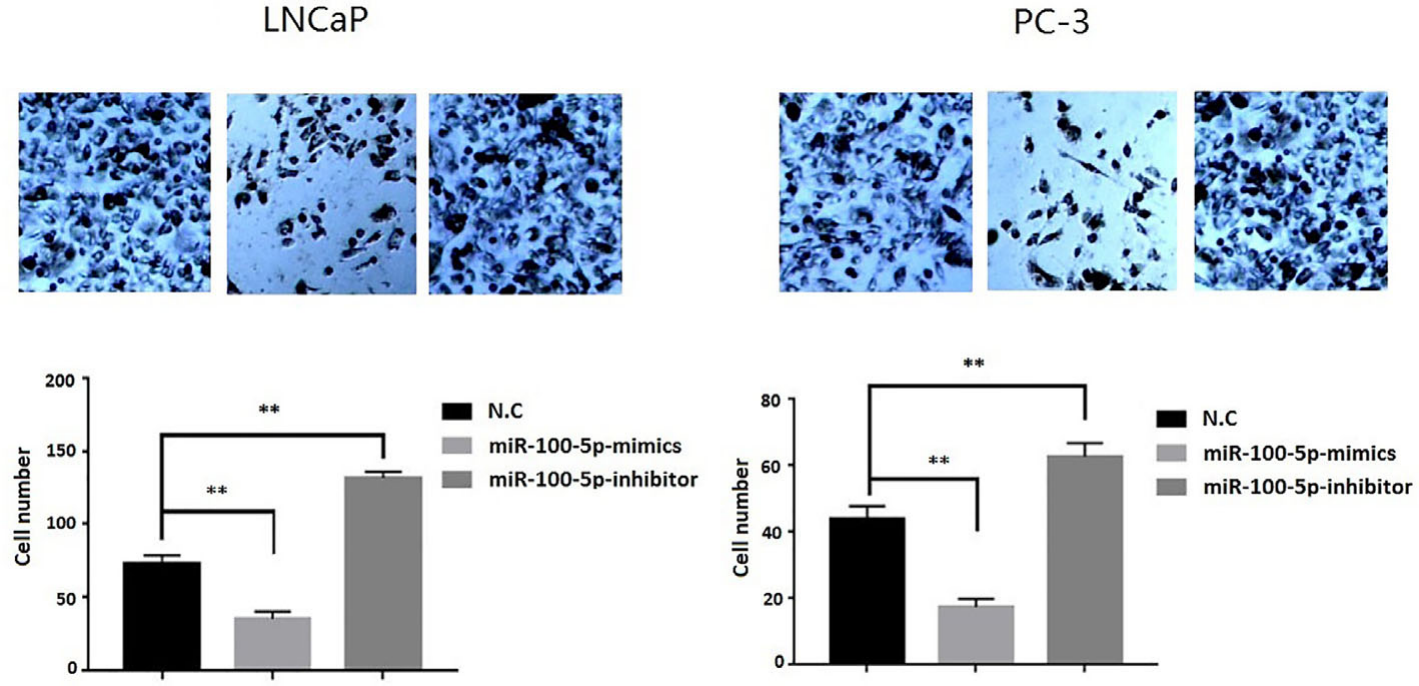
Invasion ability of PCa cells after transfection. LNCaP and PC-3 cells were transfected with miR-100-5p-mimics, inhibitor, or N.C for 48 h. (**P < 0.01).

The CCK-8 assay results indicated that the proliferation of (LNCaP and PC-3) cell lines were significantly suppressed after transfected with of miR-100-5p-mimics. In addition, colony formation was significantly weakened (P<0.01) (**Figure 3**). Compared with that of the NC-mimics group, the scratch healing rate of miR-100-5p mimics cells after transfection was significantly reduced (P<0.01), suggesting that the migration capacity of PCa cells after transfection was significantly reduced (**Figure 4**). Furthermore, the effect of miR-100-5p was examined by transwell, our results demonstrated that miR-100-5p-mimics markedly decreased numbers of cells as shown in (**Figure 5**). These results suggested that miR-100-5p acts as an onco-suppressive miRNA in PCa progression.

### mTOR Is a Novel Target Gene of miR-100-5p

**Figure 6:**
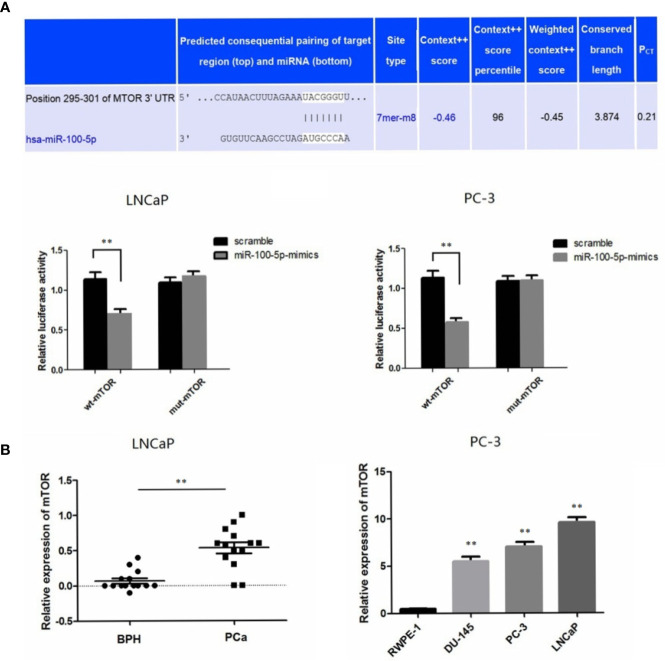
mTOR is a novel target gene of miR-100-5p. **(A)** Luciferase reporter assays demonstrated that miR-100-5p-mimic reduced WT-mTOR activity in LNCaP and PC-3 cells compared to MUT-mTOR. **(B)** qRT-PCR used to detect the expression of mTOR in serum of PCa patients and BPH and relative expression of mTOR in prostate cancer cell lines was determined by qRT-PCR compared with normal cells RWPE-1. (**P < 0.01).

A bioinformatics method was used to predict the potential target genes of miR-100-5p. By analysis in miRanda (http://www.microrna.org), mTOR was predicted to be a target gene of miR-100-5p, and there were complementary binding sites of seed sequences between miR-100-5p and the 3UTR of mTOR (**Figure 6A**). Next, the target gene of miR-100-5p was verified by luciferase reporter gene assay. miR-100-5p regulation of mTOR transcription was demonstrated in LNCaP andPC-3 cells (**Figure 6A**). The expression level of mTOR was significantly up-regulated in patients with prostate cancer. In addition, at the cellular level, the obtained results were similar. (**Figure 6B**).

### Expression of mTOR, NOX4 mRNA, and Protein

**Figure 7:**
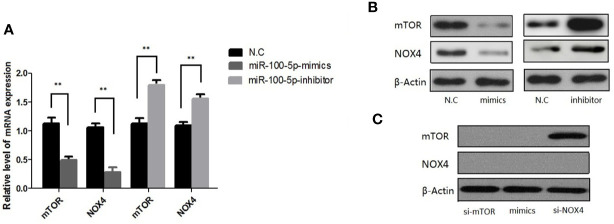
Expression of mTOR,NOX4 mRNA and protein after transfection. **(A)** The mRNA level of mTOR and NOX4 were detected after 48 h transfection by miR-100-5p-mimics, miR-100-5p -inhibitor, or N.C. **(B)** The protein level of mTOR and NOX4 were detected after 48 h transfection by miR-100-5p-mimics, miR-100-5p -inhibitor, or N.C. **(C)** The protein level of mTOR and NOX4 were detected after 48 h transfection by si-mTOR, si-NOX4, and si-mTOR+miR-100-5p-mimics, or N.C. (**P < 0.01).

qRT-PCR results showed that mTOR and NOX4 mRNA expression in LNCaP cells transfected with miR-100-5p mimics was significantly lower than that in the NC-mimics group, while it was highly expressed in miR-100-5p-inhibitor (**Figure 7A**) (P<0.01). Western blot analysis showed that mTOR and NOX4 protein expression in LNCaP cells transfected with miR-100-5p mimics was significantly lower than that in the NC-mimics group. In contrast, miR-100-5p-inhibitor significantly elevated mTOR and NOX4 protein level, as shown in **Figure 7B**. (P<0.01) We transfected LNCaP cells with si-mTOR, si-NOX4,miR-100-5p-mimic. The results showed that the protein expression of mTOR and NOX4 was significantly decreased in the cells transfected with si-mTOR and miR-100-5p-mimic compared with N.C group. On contrary, the protein expression of mTOR was no significance in group with transfected with si-NOX4, confirmed by Western blot as described in (**Figure 7C**).

### miR-100-5p Inhibits Tumor Growth *In Vivo*

**Figure 8:**
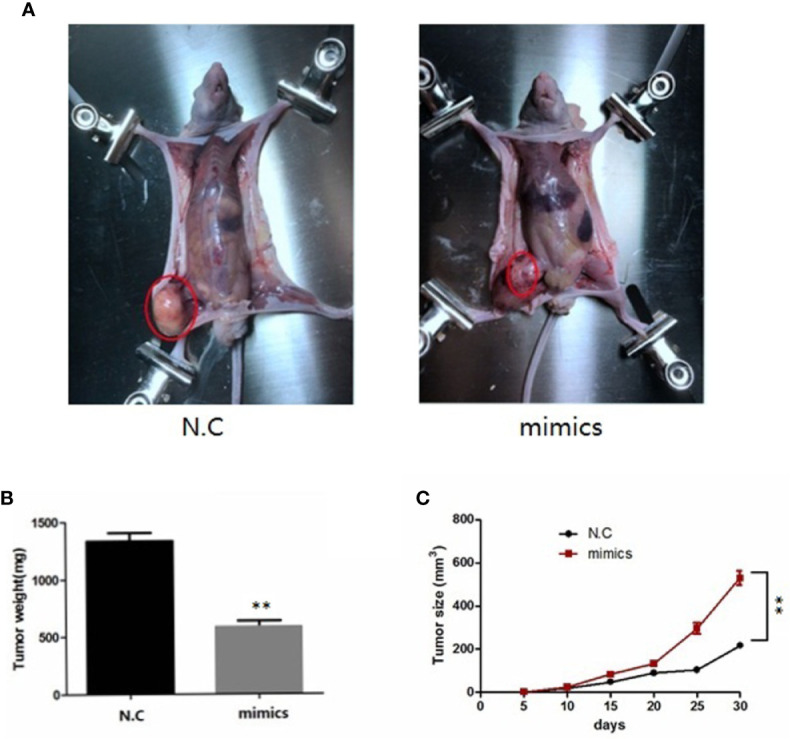
miR-100-5p has an important effect on *in vivo* tumorigenesis and clinical significance. **(A)** Six-week-old male nude mice were injected by intraosseous with miR-100-5p-mimics or N.C transfected tumor cells. **(B)** After 30 days, the mice were sacrificed and the tumor weight were measured(mg). **(C)** The tumors were measured by the ruler at 5, 10, 15, 20, 25, and 30 days, the volume of tumors were measured by the formula:V (mm3). (**P < 0.01).

**Figure 9:**
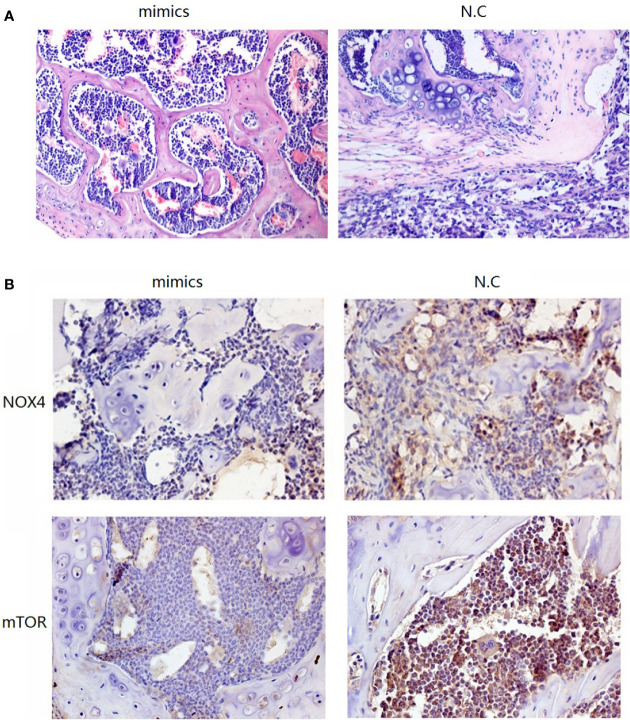
Pathological staining results. **(A)** The result of HE staining. **(B)** The results of immunohistochemical staining.

To investigate the role of miR-100-5p in the tumor growth of PCa *in vivo*, we extended our investigation by intraosseous injected tumor cells transfected with miR-100-5p mimics or N.C. We measured tumor size and found that miR-100-5p-mimic overexpressed significantly delayed tumor growth. The mice were sacrificed after 30 days. Then, the tumors were stripped and weighed. The average tumor weight in the control was significantly higher than that in the miR-100-5p-mimic group. Meanwhile, the average tumor volume in the control group was significantly larger than that in miR-100-5p-mimic group (**Figures 8A–C**). miR-100-5p could significantly inhibit the occurrence and development of osteogenic bone metastases in prostate cancer (**Figure 9A**). Immunohistochemical results showed negative expression of mTOR and NOX4 in the bone tissue of group A and positive expression in the bone tissue of group B (**Figure 9B**). Finally, these results indicated that increased miR-100-5p could inhibit PCa progression *in vivo*.

## Discussion

MiRNA was first discovered by Lee in 1993 and is a kind of ncRNA of approximately 19-24 nt (12, 13). MiRNAs can bind to multiple target mRNA 3’UTRs and regulate gene expression, resulting in abnormal expression of target genes (14, 15). Abnormal miRNA expression has been associated with the occurrence and development of tumors. Studies have confirmed that differential expression of miRNAs in tumor tissues can be used as a biomarker for early detection, typing, and prognosis of tumors (16, 17).Studies have demonstrated altered levels of miRNAs in the development of PCa, with differences between PCa patients and healthy individuals (18, 19). Studies have found that the expression of miR-100-5p is downregulated in prostate cancer, which is believed to be an important role of tumor suppressor genes in the development and progression of PCa (8, 9). It has been reported that the absence of miR-100-5p leads to the upregulation of AGO2 expression levels, thus promoting the migration, invasion and EMT of cancer cells and promoting the metastasis of prostate cancer (20).

In this study, the results of the NGS differential expression analysis showed that compared with BPH, miR-375, miR-200c-3p and miR-141-3p were upregulated, while miR-100-5p, miR-584-5p, and miR-125b-1-3p were downregulated in patients with PCa. The results of the RT-PCR analysis further confirmed the low expression of miR-100-5p in serum of PCa and LNCaP cells.

Some studies have found that miR-100-5p inhibits the proliferation, migration and invasion of tumor cells and the biological behaviors of tumors (21, 22). To further study the effects of miR-100-5p on the proliferation, invasion and migration of PCa cells, we transfected miR-100-5p mimics into PCa cells by lipofection and successfully upregulated the expression of miR-100-5p in these cells. The results of the CCK-8 proliferation assay, colony formation assay, cell scratch assay, and Transwell invasion assay showed that the upregulation of miR-100-5p could significantly inhibit the proliferation activity, migration, and invasion of tumor cells, further confirming that miR-100-5p, as a tumor suppressor gene, could inhibit the progression of PCa.

The downstream molecular pathway by which miR-100-5p inhibits the proliferation, invasion and migration of tumor cells is not completely clear. In studying the downstream target genes regulated by miR-100-5p in PCa cells, we found that the 3’UTR of miR-100-5p and mTOR has complementary seed sequence binding sites by using a bioinformatics method. Therefore, we predicted that mTOR might be a functional target gene of miR-100-5p. MIRNA modulates its target gene by binding on its complementary binding sites of mRNA 3’-UTR, and lead to inhibiting translating or degradation. In this study, the luciferase reporter gene assay verified that mTOR was a target gene of miR-100-5p. MTOR is a serine/threonine protein kinase that participates in physiological processes and pathological reactions by regulating protein synthesis and is related to the pathogenesis of cancer (23). The MTOR signaling pathway mainly regulates cell proliferation and metabolism involved in tumor development and is an important signaling pathway related to human cancer (24). In this study, we found that the expression levels of mTOR, NOX4 mRNA and protein decreased obviously in PCa cells after upregulation of miR-100-5p expression. mTOR is primarily involved in regulating the NOX4, which are involved in cancer-related signaling and are closely related to the occurrence and development of tumours (25). In the present study, we observed the effects of mTOR regulation on the NOX4. The results show that the knockdown of mTOR inhibits expression of NOX4 *in vitro*. We speculate that miR-100 curtail the invasion and migration of PCa cells by inhibiting the NOX4 through down-regulation of mTOR.

Next, nude mice in the two groups were administered by intraosseous injection with tumor cells. Group A cells were transfected with miR-100-5p mimics, and group B contained N.C transfected tumor cells (control). Pathological staining and immunohistochemical staining were performed after 1 month. HE staining of bone in group B showed a large amount of osteogenesis with tumor characteristics in the limbic cortex. The tumor cells were closely aligned, with loss of polarity and an imbalance in the nuclear to cytoplasmic ratio, and there was increased pathological nuclear fission. In group A, the trabecular structure was complete, and an obvious bone marrow cavity structure was observed. Immunohistochemical results showed negative expression of mTOR and NOX4 in the bone tissue of group A and positive expression in the bone tissue of group B. These results further confirmed that upregulation of miR-100-5p expression could significantly affected the expression of mTOR and that miR-100-5p played a role in the occurrence and development of PCa through targeted regulation of mTOR.

## Conclusion

miR-100-5p was downregulated in PCa LNCaP cell lines, and miR-100-5p affected the proliferation, migration and invasion of LNCaP cells. The mechanism is related to the downregulation of mTOR gene expression, which may become a molecular target of PCa targeted therapy in the future.

## Data Availability Statement

The original contributions presented in the study are publicly available. This data can be found here: https://www.ncbi.nlm.nih.gov/Traces/study/?acc=PRJNA673634&o=acc_s%3Aa.

## Ethics Statement

The animal study was approved by the Committees of Animal Ethics and Experimental Safety of The First Affiliated Hospital of Xi’an Medical University.

## Author Contributions

Writing—review and editing: YY. Formal analysis: S-LL. Investigation: J-JW. All authors contributed to the article and approved the submitted version.

## Funding

This study was supported by the Natural Science Basic Research Program of Shaanxi (Program No.2020JM-607).

## Conflict of Interest

The authors declare that the research was conducted in the absence of any commercial or financial relationships that could be construed as a potential conflict of interest.
